# Cardiac output measured by transthoracic echocardiography and Swan-Ganz catheter. A comparative study in mechanically ventilated patients with high positive end-expiratory pressure

**DOI:** 10.5935/0103-507X.20190073

**Published:** 2019

**Authors:** José Gorrasi, Arturo Pazos, Lucia Florio, Carlos Américo, Natalia Lluberas, Gabriel Parma, Ricardo Lluberas

**Affiliations:** 1 Cátedra de Medicina Intensiva y Centro de Tratamiento Intensivo, Facultad de Medicina, Universidad de la República - Montevideo, Uruguay.; 2 Departamento y Cátedra de Emergencia, Hospital de Clínicas, Facultad de Medicina, Universidad de la República - Montevideo, Uruguay.; 3 Cátedra de Cardiología, Hospital de Clínicas, Facultad de Medicina, Universidad de la República - Montevideo, Uruguay.

**Keywords:** Cardiac output, Hemodynamic monitoring, Echocardiography, Positive end-expiratory pressure, Pulmonary artery catheter, Respiration, artificial

## Abstract

**Objective:**

To compare cardiac output measurements by transthoracic echocardiography and a pulmonary artery catheter in mechanically ventilated patients with high positive end-expiratory pressure. To evaluate the effect of tricuspid regurgitation.

**Methods:**

Sixteen mechanically ventilated patients were studied. Cardiac output was measured by pulmonary artery catheterization and transthoracic echocardiography. Measurements were performed at different levels of positive end-expiratory pressure (10cmH_2_O, 15cmH_2_O, and 20cmH_2_O). The effect of tricuspid regurgitation on cardiac output measurement was evaluated. The intraclass correlation coefficient was studied; the mean error and limits of agreement were studied with the Bland-Altman plot. The error rate was calculated.

**Results:**

Forty-four pairs of cardiac output measurements were obtained. An intraclass correlation coefficient of 0.908 was found (p < 0.001). The mean error was 0.44L/min for cardiac output values between 5 and 13L/min. The limits of agreement were 3.25L/min and -2.37L/min. With tricuspid insufficiency, the intraclass correlation coefficient was 0.791, and without tricuspid insufficiency, 0.935. Tricuspid insufficiency increased the error rate from 32% to 52%.

**Conclusions:**

In patients with high positive end-expiratory pressure, cardiac output measurement by transthoracic echocardiography is comparable to that with a pulmonary artery catheter. Tricuspid regurgitation influences the intraclass correlation coefficient. In patients with high positive end-expiratory pressure, the use of transthoracic echocardiography to measure cardiac output is comparable to invasive measures.

## INTRODUCTION

Hemodynamic monitoring of critical patients is useful to characterize the state of hemodynamics, make the diagnosis, and guide treatments. It allows us to characterize oxygen transport to tissues and oxygen metabolism.^(1)^ There are many ways to measure cardiac output, such as continuous measurements, blood flow measurements, thermodilution measurements, and pulse wave variability measurements. The standard method is pulmonary thermodilution performed with a pulmonary artery catheter (PAC). Swan and Ganz implemented it for clinical use in the 1970s.^(2)^

The use of PAC has been criticized because it is an invasive method requiring right heart and pulmonary artery catheterization and is therefore prone to possible complications; because interpretation of the results and its clinical application are related to the knowledge and training of the operator; and because all the above increase the iatrogenic load.^(3,4)^ Among the noninvasive cardiac output measurement methods, transthoracic echocardiography (TTE) has been reported as a hemodynamic assessment tool.^(5)^ Focused cardiac TTE can help assess global cardiac function to guide less invasive treatments.^(6)^ Transthoracic echocardiography provides valuable information on diastolic function, cardiac structures, regional motility, and valve function.^(7-9)^ There are sufficient data and studies comparing TTE and standard hemodynamic assessment in outpatients and mechanically ventilated patients.^(10)^ Mechanically ventilated patients present changes that could modify cardiac output measurement, including an inadequate ultrasonic window; positive pressure and positive end-expiratory pressure (PEEP), which could modify hemodynamic conditions; and the presence of tricuspid regurgitation. Mechanical ventilation and PEEP modify the determinants of cardiac function, possibly resulting in the inadequate correlation between different cardiac output measurement methods.^(11,12)^ Tricuspid regurgitation is common in mechanically ventilated patients and might influence cardiac output measurements.^(13-15)^

Our hypothesis is that cardiac output measurement by TTE is comparable to invasive PAC measurements in mechanically ventilated patients with high PEEP.

The present study aims to compare cardiac output measurements performed with TTE and PAC in mechanically ventilated patients with high PEEP.

## METHODS

This is a comparative study of cardiac output measurement in a convenience sample of patients admitted to Intensive Care, where hemodynamic variables were studied by two methods (TTE and PAC) at different PEEP levels.

Between January 2011 and December 2012, mechanically ventilated patients admitted to the Intensive Care Center at the *Hospital de Clinicas* who underwent PAC performed by the treating medical team were studied. Indications for PAC placement were cardiogenic shock, septic shock, advanced heart failure with peripheral hypoperfusion, shock of unclear etiology, severe respiratory failure, and post-cardiac surgery with postoperative shock. Nine patients were excluded because of an inadequate ultrasonic window, severe hemodynamic instability, severe arrhythmia, or aortic or mitral valve disease. The quality of the acoustic window was graded as follows: 0: no transthoracic echocardiographic image was obtained; 1: the endocardium of the ventricles could not be fully visualized (poor visualization of the heart valves and/or the great vessels); 2: short segments of the endocardium were not completely visualized (partial visualization of the heart valves and/or the great vessels); 3: complete visualization of both ventricles (complete visualization of the heart valves and/or the great vessels).^(16)^

The study was conducted according to the Declaration of Helsinki's ethical principles for medical research involving human subjects. The study was approved by the Medical Ethics Committee of the *Hospital de Clínicas*. Informed consent was signed by relatives. Patients were mechanically ventilated and treated with sedation and analgesia using routine doses of midazolam and fentanyl.

Echocardiographic measurements were performed with a Siemens Acuson ultrasound system by cardiologists with 3 years of experience performing echocardiography in critical patients. Echocardiographic variables followed the standards of the American Society of Echocardiography (ASE).^(17)^

The Doppler-estimated cardiac output in the apical five-chamber view was derived from systolic volume using the time-velocity integral (TVI) of the flow through the left ventricular outflow tract (LVOT), the LVOT diameter, and heart rate during the imaging study. The aortic TVI was recorded from the apical view by placing the Doppler sample volume in the LVOT below (5mm proximal) the level of the aortic valve. LVOT diameter was measured on the long parasternal axis. Cardiac output was calculated using the following formula

Heartbeat volume = TVI (cm) (cross-sectional area)

Cross-sectional area = π (LVOT diameter/2)^2^

where π is equal to 3.1416

Heartbeat volume = π (LVOT diameter/2)^2^(cm^2^) TVI (cm) = cm^3^ or mL

Cardiac output = heart rate (beats per minute) x TVI (cm) x 3.1416 x (LVOT diameter (cm)/2)^2^(cm^2^) = mL per minute

Cardiac dimensions and ventricular function were measured according to ASE guidelines.^(17,18)^ Left ventricular ejection fraction (LVEF) was evaluated by Simpson's method. Tricuspid regurgitation was evaluated by continuous Doppler TTE according to ASE guidelines. The severity of tricuspid regurgitation was classified as mild, moderate, or severe.^(18)^

### Swan-Ganz measurements

Invasive measurements were performed with a PAC (Biosensor^®^). The catheter was placed by Intensive Care doctors in charge of the patient; the jugular and subclavian central venous lines were used without complications. The procedure was performed using the cuff method to confirm pulmonary wedge pressures and chest X-ray control. The pulmonary catheter was connected to a General Electric Solar^®^ monitor for pressure recording and cardiac output measurement by thermodilution. Cardiac output was measured by pulmonary thermodilution with the PAC. Measurements were performed at the end of expiration, taking the average of five consecutive measurements but taking out the smallest and largest measurements. Measurements with variability > 10% were discarded.^(19)^ Pulmonary thermodilution was performed by injection of 10mL saline into the proximal end of the PAC, verifying the presence of a thermodilution curve.^(20-22)^ Cardiac output was computed on a General Electric Solar^®^ monitor. Pressures were measured in mmHg, with the zero reference at the level of the mid-thorax. Measurements included diastolic atrial pressure (DAP), pulmonary artery systolic pressure (PASP), pulmonary artery mean pressure (PAMP), pulmonary artery diastolic pressure, and pulmonary capillary wedge pressure (PCWP). Pulmonary vascular resistance (PVR) in Wood units was calculated using the equation PVR = (PAMP - PCWP)/CO; systemic vascular resistance (SVR) was calculated by the equation SVR = (mean arterial pressure - DAP)/cardiac output. Both are expressed as dynes.s.cm^-5^ and dynes.s.cm^-5^.m^-2^.

### Effect of positive end-expiratory pressure

Patients were studied at three PEEP levels, 10cmH_2_O, 15cmH_2_O, and 20cmH_2_O, allowing 5 minutes of stabilization between measurements. When the patient was at one of these levels, PEEP was not decreased because it was considered part of the treatment. At each PEEP level, cardiac output was measured by TTE and by PAC consecutively, performing 5 cardiac output measurements with PAC and 3 with TTE to obtain each cardiac output value.

At each PEEP level, a pair of cardiac output measurements were obtained by PAC and TTE for each patient. When it was not possible to obtain a stable condition, hemodynamic measurements were not recorded. There were no adverse events during the PEEP protocol.

### Statistics

Values are expressed as mean, standard deviation (SD), and range. Categorical data are expressed as frequencies and percentages. Normality was tested with the Kolmogorov-Smirnov test. The Kruskal-Wallis test was used to compare continuous data. The agreement between the methods was assessed with the intraclass correlation coefficient (ICC) at each PEEP level; ICC > 0.75 was considered excellent, with p < 0.05 deemed significant. The limits of agreement (LoA) and error between both methods, and the SD of the differences (accuracy), were studied with the Bland-Altman plot (plot of the difference against the mean of the measurements). The error rate was calculated as two times the SD of the mean of all measurements and is expressed as a percentage. Accuracy was calculated as the SD of the differences between both methods, and dispersion as the range of the limits according to the differences between the two methods. The coefficient of variability was calculated by dividing the SD of the measurements by the mean of the measurements for each patient as reported by PAC and TTE. The effect of temperature on cardiac output was evaluated by simple linear regression. The variability of TVI in cardiac output measurement was assessed with cardiac output measured by PAC at cardiac output values < 5L/min or ≥ 5L/min and is expressed as mean, SD, and range.

## RESULTS

Twenty-five patients were studied, of whom we could include 16 patients. Patients who underwent PAC at the discretion of the treating team were included. Clinical characteristics of the patients included in the study were age 60 ± 15 years, time on mechanical ventilation 4 ± 2 days, and mortality 31% (Table 1). The diagnoses were post-cardiac surgery (n = 3), sepsis (n = 6), acute myocardial infarction (n = 3), acute coronary syndrome (n = 2), and heart failure (n = 2).

**Table 1:** Population characteristics

Variable	
Age (years)	60 ± 15
CO* (L/min)	6.7 ± 2.8
Heart rate (bpm)	102 ± 15
DAP* (mmHg)	18 ± 6
Mechanical ventilation (days)	4 ± 2
PASP* (mmHg)	42 ± 13
PCWP* (mmHg)	22 ± 5
APACHE II	19 ± 7
Mortality (%)	31
Ventilatory parameters	
PaO_2_/FIO_2_	226 ± 72
Ventilation mode	VCV
Plateau pressure (cmH_2_O)	27 ± 5
Semistatic respiratory compliance (mL/cmH_2_O)	29 ± 9
Respiratory rate (rpm)	16 ± 4

DAP - diastolic atrial pressure; PASP - pulmonary artery systolic blood pressure; PCWP - pulmonary capillary wedge pressure; APACHE II - Acute Physiology and Chronic Health Evaluation II; PaO_2_/FIO_2_ - arterial partial pressure/inspired fraction of oxygen; VCV - volume-controlled ventilation.

* Measured by pulmonary artery catheter. Values are expressed as mean and SD.

Of the 16 patients, 44 pairs of cardiac output measurements were obtained. In 14 patients, 14 pairs of cardiac output measurements by TTE and PAC were performed at the three PEEP levels (14 by 3 = 42 pairs); in addition, in one patient, a pair of measurements were performed with PEEP 10cmH_2_O; and in another patient, a pair of measurements were performed with PEEP 15cmH_2_O; so a total of 44 measurement pairs were completed.

To evaluate the variability for each diagnostic method, we evaluated the range of cardiac output values (maximum minus minimum) and the percentage of measurement pairs contributing to all measurement pairs. We found that the pairs of sepsis measurements totaled 41%, and the range of cardiac output measurements was 8.30L/min. The measurement pairs in post-cardiac surgery patients were 16%, with a range of cardiac output measurements of 6.7L/min. The measurement pairs of acute coronary syndrome (ACS) totaled 14%, with a range of cardiac output measurements of 0.80L/min. The measurements pairs of heart failure totaled 14%, with a range of cardiac output measurements of 4.90L/min. The measurement pairs of acute myocardial infarction (AMI) totaled 16%, with a range of cardiac output measurements of 2.20L/min. Temperature had no effect on cardiac output values (R^2^ = 0.073, p = 0.183).

All patients were mechanically ventilated and treated with sedation and analgesia. With PEEP 10cmH_2_O, semistatic compliance was 29 ± 9mL/cmH_2_O, PaO_2_/FIO_2_ was 226 ± 72, plateau pressure 27 ± 5cmH_2_O, and respiratory rate 16 ± 4rpm.

Regarding treatment with vasopressors and inotropic agents, 36% required norepinephrine and 21% dobutamine. The mean cardiac output measured by TTE was 7.0 ± 2.6L/min, while that obtained with PAC was 6.6 ± 2.9L/min out of all measurements. There were no significant differences in cardiac output measured by TTE and PAC at any PEEP level (Table 2). The coefficient of variability of cardiac output measurement by PAC was 6% and by TTE 9%.

**Table 2:** Bland-Altman results for cardiac output

PEEP (cmH_2_O)	n	CO (TTE) (L/min)	CO (PAC) (L/min)	ME ± SD (L/min)	LoA (L/min)	Average sum of both methods&	Dispersion (L/min)	Accuracy (L/min)	Error rate %
10	15	7.4 ± 2.7; (3.3 -11.0)	6.9 ± 2.9; (3.1 - 11.8)	0.46 ± 1.55	-2.58 a 3.50	7.20 ± 2.73	5.62	1.55	43
15	15	6.6 ± 2.4; (3.3 - 11.1)	6.0 ± 2.8; (3.1 - 12.1)	0.56 ± 1.17	-1.73 a 2.86	6.36 ± 2.53	4.03	1.17	37
20	14	6.9 ± 2.5; (3.0 - 12.4)	6.7 ± 3.0; (3.1 - 13.0)	0.29 ± 1.64	-2.93 a 3.52	6.80 ± 2.71	6.15	1.64	48
Total	44	7.0 ± 2.6; (3.0 - 12.4)	6.6 ± 2.9; (3.1 - 13.0)	0.44 ± 1.43	-2.37 a 3.25	6.79 ± 2.65	5.62	1.43	42

PEEP - positive end-expiratory pressure; CO - cardiac output; TTE - transthoracic echocardiography; PAC - pulmonary artery catheter; ME - mean error (average difference between methods); SD - standard deviation; LoA - limits of agreement. Dispersion is the range of limits of agreement of the differences. Accuracy is equal to the SD of differences, i.e., of the average maximum and minimum of the sum of both methods (L/min). Mean error, mean difference between methods ± 1.96 standard deviation;

& mean ± standard deviation. Error rate (100 multiplied by 2 standard deviations divided by the average of cardiac output values). n, number of measurement pairs.

Regarding the presence of tricuspid regurgitation, with PEEP 10cmH_2_O, tricuspid regurgitation was detected in 10 of 15 measurements (67%); with PEEP 15cmH_2_O, in 8 of 15 measurements (53%); with PEEP 20cmH_2_O, in 6 of 14 measurements (43%). In total, tricuspid regurgitation was detected in 24 of 44 cardiac output measurements (55%).

The ICC for all cardiac output measurements by TTE and PAC was 0.919 (95% confidence interval - 95%CI, 0.851 - 0.956; n = 44); for PEEP 10cmH_2_O, 0.901 (95%CI, 0.706 - 0.936; n = 15); for PEEP 15cmH_2_O, 0.947 (95%CI, 0.841 - 0.982; n = 15); for PEEP 20cmH_2_O, 0.908 (95%CI, 0.713 - 0.970; n = 14) (p < 0.001) (Figure 1a). With tricuspid regurgitation, ICC was 0.791 (95%CI, 0.576 - 0.904; n = 24); without tricuspid regurgitation, ICC was 0.935 (95%CI, 0.848 - 0.973; n = 20).

**Figure 1a:**
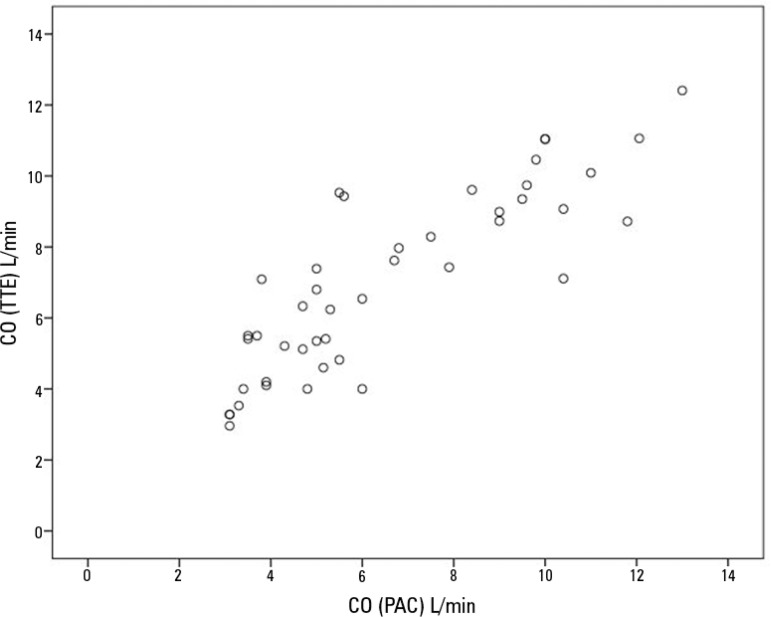
Cardiac output correlation for all measurements. ICC = 0.919 (95% CI, 0.851 - 0.956), p < 0.001, n = 44. CO - cardiac output; TTE - transthoracic echocardiography; PAC - pulmonary artery catheter.

Bland-Altman analysis for all cardiac output measurements showed a bias of 0.44L/min, with LoA between -2.37L/min and 3.25 L/min. Accuracy was 1.43L/min, error rate 42%, and dispersion 5.18L/min (Figure 1b). With different PEEP levels, the values of bias, LoA, and error rate did not vary significantly (Table 2 and Figure 1b). Tricuspid regurgitation increased the error rate from 32% to 52% (Table 3).

**Figure 1b:**
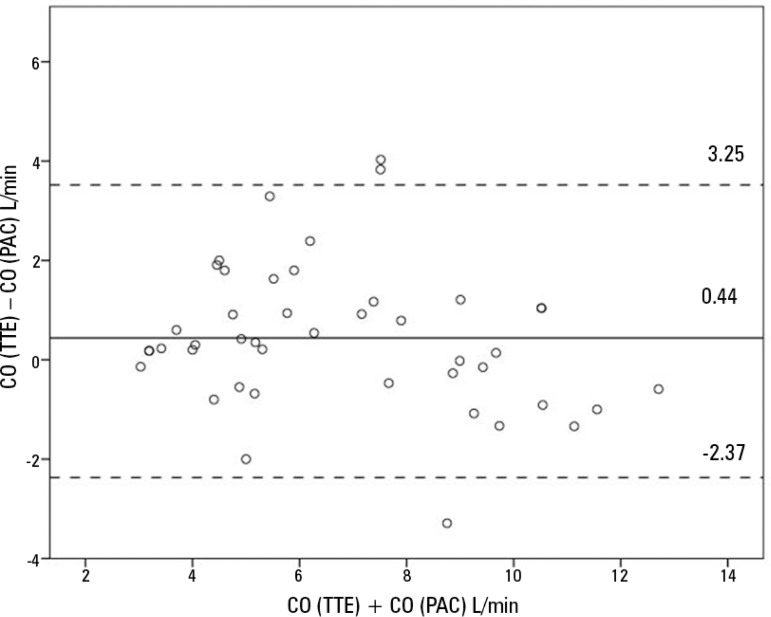
Bland-Altman plot for all cardiac output measurements. Mean error: 0.44 L/min. Limits of agreement: -2.37 L/min to 3.25 L/min. CO - cardiac output; TTE - transthoracic echocardiography; PAC - pulmonary artery catheter.

**Table 3:** Effect of tricuspid insufficiency on cardiac output measurement

	Without tricuspid insufficiency (n = 20)*		With tricuspid insufficiency (n = 24)*		Difference (L/min)**	
	TTE	PAC	TTE	PAC	TTE	PAC
Average CO (L/min)	7.40 ± 2.51	6.95 ± 2.94	6.68 ± 2.59	6.25 ± 2.84	0.72**	0.69**
Average CO of both methods (L/min)	7.17± 2.68; (13 - 4)		6.47 ± 2.59; (12 - 3)		0.71**	
Mean error (L/min)	0.45 ± 1.14		0.43 ± 1.67		0.02**; 0.53&	
Error rate (%)	32		52			

TTE - transthoracic echocardiography; PAC - pulmonary artery catheter; CO - cardiac output. Values are expressed as the mean ± standard deviation; maximum - minimum.

* Number of measurements.

** difference of means;

& difference of standard deviation. Relationship of standard deviation with tricuspid insufficiency divided by the standard deviation without tricuspid insufficiency = 1.67/1.14 = 1.43. The percentage increase of standard deviation with tricuspid insufficiency is 100 (1.67 - 1.14)/1.14 equal to 46. The error rate is equal to 100 by the standard deviation by 2/cardiac output average of both methods.

We found a TVI range dispersion from 9cm to 34cm, which partly explains the dispersion of cardiac output values. When pulmonary thermodilution cardiac output was > 5L/min, TVI was > 16 cm (range 16 cm to 34cm, 24 ± 5). When cardiac output was < 5L/min, the TVI range was 9 cm to 22cm (18 ± 4cm).

Left ventricular ejection fraction was mild to moderately decreased but did not change between different PEEP levels. A significant correlation was found between LVEF and cardiac output measured by TTE (r = 0.373) and PAC (r = 0.562). No patent foramen ovale was detected at any PEEP level. PCWP measured by PAC at all PEEP levels was 22 ± 5mm Hg; systemic vascular resistance index (SVRI) was 1569 ± 496 dynes.s.cm^-5^.m^2^; and pulmonary vascular resistance index (PVRI) 269 ± 154 dynes.s.cm^-5^.m^2^; the values did not vary significantly between different PEEP levels.

## DISCUSSION

Our study found it was possible to perform noninvasive cardiac output measurement by TTE in mechanically ventilated patients with high PEEP. Its values were adequately correlation with the values from PAC pulmonary thermodilution. The mean error was 0.44L/min, with LoA of ± 2.81L/min and an error rate of 42%. When we studied the presence of tricuspid regurgitation, we found it was correlated with a decrease in the ICC and an increase in the error rate.

Determination of cardiac output by Doppler echocardiography has been validated in other studies in different patient populations with spontaneous ventilation or mechanical ventilation.^(23-27)^ Our study adds information to the literature on this topic.^(23-29)^ The mean error, LoA, and error rate in our study are comparable to those in other studies of cardiac output measurement by TTE. The mean error of previous studies ranges between 0.03 and 0.75 L/min, LoA between ± 0.57 and ± 2.87L/min, and error rate between 11% and 69%. The error rate of an acceptable method for cardiac output measurement should be approximately 30%.^(30-32)^ The error rate reported in previous comparative studies of cardiac output has varied between 11% and 69%, in agreement with the error rate in our study.^(23,24,33-35)^

The difference in agreement between the TTE and PAC methods can be explained by several factors: increased gas between the transducer and the heart that would modify the alignment between measurements, changes in cardiac output determined by the effect of positive pressure on hemodynamics, venous return, and afterload.^(36-39)^ Positive end-expiratory pressure, when recruiting alveolar units, causes an increase in the pulmonary volume interposed between the transducer and the heart, thus reducing the quality and sharpness of the echocardiographic image obtained. Moreover, PEEP modifies the conditions of cardiac filling and afterload, which could modify the cardiac physiological condition by changing the pattern of cardiac filling and ventricular ejection time.^(40-43)^ Previously,^(44-49)^ cardiac output has ranged between 2.5L/min and 12.0L/min; in our study, cardiac output ranged between 3L/min and 13L/min. This can be explained by the heterogeneity attributable to the different conditions included, such as sepsis and acute coronary syndrome, as well as temperature values and the limitations of pulsed Doppler for detecting high cardiac output values when the blood flow velocity is > 2m/s. Sepsis patients and post-cardiac surgery patients showed the greatest variability in cardiac output while contributing to a significant percentage of the number of measurement pairs. This element undoubtedly modified the SD of the measurements, so the error rate calculated was greater. In the reported studies, this element was not analyzed, even in heterogeneous patient populations.^(26,29,49)^ The presence of a patent foramen ovale can induce error in cardiac output measurement by causing an intracardiac shunt; in our study, we did not find a patent foramen ovale. A patent foramen ovale is uncommon even with acute cor pulmonale.^(50)^ Tricuspid regurgitation has been observed in mechanically ventilated patients, reaching 70% of them in some series.^(51,52)^ The effect of tricuspid insufficiency on cardiac output measurement by PAC has been considered relevant, especially in severe tricuspid regurgitation.^(52)^ In our study, tricuspid regurgitation was detected, although there was no statistically significant change between cardiac output values by the two methods. However, tricuspid regurgitation was correlated with a decrease in the ICC and an increase in the error rate. We attribute the latter to the increase in the SD because of the wide range of cardiac output values in the presence of tricuspid regurgitation. Severe tricuspid regurgitation causes an underestimation of cardiac output by thermodilution and an overestimation of cardiac output by echocardiography.^(13,51,52)^ As a result, there is an increase in error and LoA when we compare thermodilution with echocardiography, as shown by Balik et al. using transesophageal echocardiography.^(13)^ Of the previous studies, only Temporelli et al. examined tricuspid regurgitation, finding tricuspid regurgitation in 50% had severe tricuspid regurgitation in 15%.^(35)^

This study has several limitations. First, the sample size was small compared to previous studies, where n was between 18 and 48 patients. Second, the sample size was not calculated for the type of study, although none of the previous studies did so. Third, the sample heterogeneity, with different pathophysiological conditions (cardiogenic shock and distributive shock), likely increased the range of cardiac output values and their dispersion. Sepsis patients and post-cardiac surgery patients had the greatest variability in cardiac output. Fourth, when hemodynamic measurements were performed, we were not blind to changes in PEEP or to the results of both techniques at the time of obtaining them. Fifth, we must consider the errors in Doppler cardiac output measurement in values close to 12L/min, when blood flow rates are > 2m/s. This point has been discussed in studies where cardiac output was increased with dobutamine; in this situation, it was found that the laminar blood flow profile is maintained and the Doppler cardiac output correlates with the thermodilution method.^(53)^ Sixth, the position of the sample volume in the LVOT and the measurement accuracy of the area of the LVOT could be a source of error in high-cardiac output situations when blood flow energy and the mechanical properties of the wall can cause changes in actual flow area.^(54,55)^ Stewart et al. found that when blood flow was increased experimentally, TVI increased more than the diameter of the vascular cross-section area.^(56)^ Seventh, it was not an original study, since there are previous studies comparing both methods, including a recently published one comparing cardiac output measurement in mechanically ventilated patients performed by Intensive Care doctors.^(29)^

## CONCLUSIONS

In our sample of mechanically ventilated patients with high positive end-expiratory pressure, cardiac output measurement by transthoracic echocardiography was comparable to cardiac output measured by pulmonary artery catheter, with a mean error and an error rate within the reported limits. Therefore, transthoracic echocardiography can measure cardiac output reliably. The presence and effect of tricuspid regurgitation on cardiac output measurement should be analyzed in a larger and more homogeneous series of patients.
